# Bidirectional teaching between lightweight multi-view networks for intestine segmentation from CT volume

**DOI:** 10.1117/1.JMI.12.2.024003

**Published:** 2025-03-31

**Authors:** Qin An, Hirohisa Oda, Yuichiro Hayashi, Takayuki Kitasaka, Aitaro Takimoto, Akinari Hinoki, Hiroo Uchida, Kojiro Suzuki, Masahiro Oda, Kensaku Mori

**Affiliations:** a Nagoya University, Graduate School of Informatics, Nagoya, Japan; b University of Shizuoka, School of Management and Information, Shizuoka, Japan; c Aichi Institute of Technology, School of Information Science, Toyota, Japan; d Nagoya University, Graduate School of Medicine, Nagoya, Japan; e Aichi Medical University, Department of Radiology, Nagakute, Japan; f Nagoya University, Information Technology Center, Nagoya, Japan; g National Institute of Informatics, Research Center for Medical Bigdata, Tokyo, Japan

**Keywords:** intestine segmentation, semi-supervision, computer-aided diagnosis, pseudo-label

## Abstract

**Purpose:**

We present a semi-supervised method for intestine segmentation to assist clinicians in diagnosing intestinal diseases. Accurate segmentation is essential for planning treatments for conditions such as intestinal obstruction. Although fully supervised learning performs well with abundant labeled data, the complexity of the intestine’s spatial structure makes labeling time-intensive, resulting in limited labeled data. We propose a 3D segmentation network with a bidirectional teaching strategy to enhance segmentation accuracy using this limited dataset.

**Method:**

The proposed semi-supervised method segments the intestine from computed tomography (CT) volumes using bidirectional teaching, where two backbones with different initial weights are trained simultaneously to generate pseudo-labels and employ unlabeled data, mitigating the challenge of limited labeled data. Intestine segmentation is further complicated by complex spatial features. To address this, we propose a lightweight multi-view symmetric network, which uses small-sized convolutional kernels instead of large ones to reduce parameters and capture multi-scale features from diverse perceptual fields, enhancing learning ability.

**Results:**

We evaluated the proposed method with 59 CT volumes and repeated all experiments five times. Experimental results showed that the average Dice of the proposed method was 80.45%, the average precision was 84.12%, and the average recall was 78.84%.

**Conclusions:**

The proposed method can effectively utilize large-scale unlabeled data with pseudo-labels, which is crucial in reducing the effect of limited labeled data in medical image segmentation. Furthermore, we assign different weights to the pseudo-labels to improve their reliability. From the result, we can see that the method produced competitive performance compared with previous methods.

## 1 Introduction

**Fig. 1:**
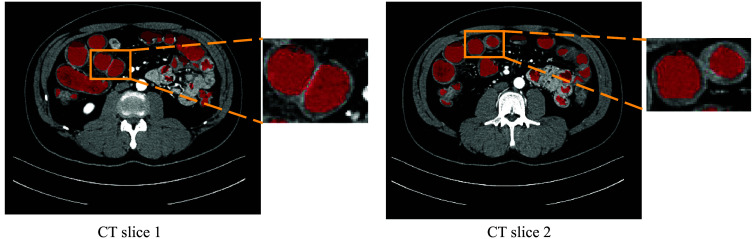
Example of CT slices. We enlarge some regions in the yellow boxes and see some contact in the intestine wall although just segmenting lumen regions can avoid this contact. In the enlarged parts, the red and light regions denote the lumen and wall, respectively.

Intestine obstruction^1^^–^^3^ is a severe disease involving mechanical or functional intestine blockage, often resulting in intense abdominal pain, vomiting, and distention. Computed tomography (CT) is an important imaging method that can help clinicians diagnose diseases by providing more detailed imaging of the intestines. However, it is time-consuming for clinicians to check a patient’s CT volume. Computer-assisted diagnosis systems can assist clinicians in accurately identifying obstructions within the CT volume. However, the intestine is long and folded in the abdomen, which will cause most of the contact in the intestine wall. Therefore, we aim to develop a segmentation method that extracts the intestine lumen, including the small and large intestine lumens, to avoid most contact and help clinicians better understand the intestine’s structure. In the paper, the word “intestine segmentation” denotes the small and large bowel lumen segmentation. The CT slices in Fig. 1 show that just segment lumen regions can avoid most contact.

For organ segmentation, numerous methods^4^^–^^6^ have been explored by researchers. Most of these methods rely on pixel-level labeled data. However, medical image annotations at the pixel level are more difficult than those of natural images because clinicians with specialized anatomical knowledge need to do the former. Given the limited labeled dataset, the effective utilization of unlabeled data becomes crucial for medical image segmentation. Based on that, the segmentation of the intestine presents more challenges due to its elongated, tubular morphology, and intricate spatial configuration, setting it apart from larger organs such as the liver. At present, several methods have been employed for intestine segmentation. Rajamani et al.^7^ proposed using a region growing method to segment the intestine regions from CT volumes. Zhang et al.^8^ also employed a region growing combined with the pre-trained probability map to segment the colon. Frimmel et al.^9^ proposed using the centerline to segment the colon. Bert et al.^10^ proposed an adaptive 3D region-growing algorithm to segment the colon. The three previous methods segment the colon from CT volumes based on the region-growing method. Shin et al.^11^ proposed adding cylindrical topological constraint into 3D U-Net to segment small bowel. Oda et al.^12^ employed an improved 3D U-Net to segment intestines (including small and large bowel) from CT volumes. The two methods employed the fully supervised method for the intestine segmentation task. The intestine has a complex spatial structure and low contract between the surrounding tissue, which increases the difficulty of segmenting the intestines from CT. Compared with the region-growing method, fully supervised methods can extract comprehensive semantic features of the intestine from CT volumes to achieve better segmentation results. However, fully supervised methods require a large amount of labeled data, which is time-consuming and difficult. Therefore, we propose using an improved semi-supervised method to segment the intestine from CT volumes. These methods just rely on fully supervised learning or hand-crafted features. Furthermore, the intestine has a complex spatial structure and low contract between the surrounding tissue, which increases the difficulty of segmenting the intestines from CT.

Semi-supervised segmentation methods offer a pragmatic compromise between supervised and unsupervised learning by harnessing both labeled and unlabeled data. Their key advantage over purely supervised learning lies in their ability to leverage a larger pool of unlabeled data, often more readily available, to enhance model training. A noteworthy example of such a semi-supervised segmentation method is cross pseudo-supervision (CPS).^13^ CPS utilizes a combination of labeled and unlabeled data, using labeled data to generate pseudo-labels for unlabeled data. By incorporating information from both labeled and unlabeled data, CPS enhances the model’s adaptability and robustness, ultimately leading to more accurate and reliable segmentation results. In recent research, several semi-supervision segmentation methods^14^^,^^15^ have attracted researchers’ attention and applied them to organ segmentation tasks. Pseudo-labels,^16^ mean teacher (MT) models,^17^ and entropy minimization (EM),^18^ all of these methods based on semi-supervision, have achieved good performance in some organ segmentation tasks. However, it is notable that most existing intestine segmentation methods have yet to use the advantages of semi-supervised segmentation techniques, which effectively utilize both labeled and unlabeled data. Therefore, this work introduces an innovative approach designed to harness the potential of unlabeled data for the specific task of intestine segmentation.

We propose a novel segmentation network called a lightweight multi-view symmetrical network (LMVS-Net) to learn more intestine information by effectively extracting multi-scale semantic information, which can improve the model’s learnability and solve the challenge caused by complex structure and low contrast. Furthermore, inspired by the CPS,^13^ we incorporate the bidirectional teaching strategy with the new backbone, LMVS-Net, to utilize the unlabeled data to decrease the influence of limited labeled data common in organ segmentation tasks. The proposed method was developed based on our SPIE conference’s method (MVS-Net).^19^ Based on MVS-Net, we employ the stacks of small-sized convolutional kernels rather than large convolutional kernels to reduce the number of model parameters while maintaining segmentation accuracy. In addition, we utilize distance map weight in the loss function. In addition to the optimization of the methods, we also increase experiments, such as using different numbers of labeled data to validate the method and the ablation study about loss functions to make the research more comprehensive.

The framework trains two networks with the same structure but different initial weights simultaneously, taking labeled and unlabeled data as input. The labeled data are used to calculate the supervision loss, which guides the training process. The unlabeled data are utilized to calculate the loss value with pseudo-labels generated by the predictions of the two sub-networks. This is a strategy that makes the network use large-scale unlabeled datasets by generating pseudo-labels in semi-supervision methods. However, the pseudo-labels may be unreliable because they rely on the prediction of the network, especially in the early stages of training. In general, the prediction of the central parts, which are not adjacent to the background of the organ, is reliable. The peripheral parts, which are adjacent to the background, are unreliable due to the peripheral parts having complex situations, such as contact with other tissues or organs. Thus, we assume that the predictions of the central parts are more reliable than those of the peripheral parts, and this reliability is represented in terms of the closest distance between the closest background and each foreground part. To decrease the influence of the unreliability of the pseudo-labels during loss calculation, we generate a distance map according to the Euclidean distance transform of pseudo-labels and assign different weights to different regions calculated by the distance between the closest background regions to each foreground. Furthermore, the intestine is a tubular-shaped organ. For such organs, centerline-based information can be utilized as their shape-based feature. We employ not only the conventional Dice^20^ but also the clDice loss,^21^ which can use centerline information to guide the training.

This paper proposes that the LMVS-Net combined with bidirectional teaching be used to segment the intestine from CT volumes. The contributions of this paper are summarized as:

1. We present the lightweight multi-view model, LMVS-Net, for the intestines segmentation task.
2. We generate a distance weight map from the pseudo-labels to reduce the influence of potentially unreliable exist in pseudo-labels.

## 2 Method

### 2.1 Overview

The proposed method segments the intestine regions from a CT volume. In the method, we simultaneously train two LMVS-Nets having different initial weights. The training data consist of limited labeled data and large-scale unlabeled data. Consequently, two loss functions are defined, one for labeled data called supervision loss and the other for unlabeled data called unsupervised loss. In detail, the loss value for labeled data is calculated to update the models’ parameters by supervision loss. The bidirectional teaching strategy generates pseudo-labels for unlabeled data for updating the parameters by unsupervised loss.

**Fig. 2:**
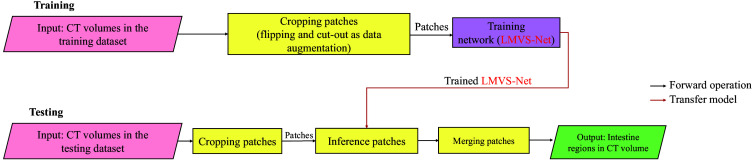
Flowchart of the intestine segmentation method in training and testing. In the training, we use CT volumes as input with some data augmentation to train a segmentation model. In the testing, we also crop the CT patches and then use the trained model to infer the patches. Finally, we merge them as the final output.

In the training, we crop the CT patches with size 256×256×16  voxels and stride as 128×128×8  voxels from CT volumes and randomly choose these patches as mini-batches to train the segmentation model. We apply flipping and cut-out^22^ operations as data augmentation before training. In the testing, we also crop the CT patches from CT volumes by overlapped sliding windows with size 256×256×16  voxels and the stride 128×128×8  voxels to infer the segmentation result by a trained segmentation model. Finally, we merge the results of those patches with the same size as the CT volume to generate the output of the testing. We use the average of predicted probability for the overlapped regions as the final result. Figure 2 shows the flowchart of the intestine segmentation method.

### 2.2 Lightweight Multi-view Symmetric Network

The LMVS-Net enhances its learning capability by employing various sizes of convolutional kernels to capture multi-view features from various perceptual fields. Therefore, the model is called multi-view from the feature level. In addition, it effectively reduces the number of network parameters by stacking small-sized convolutional kernels instead of using large ones.

**Fig. 3:**
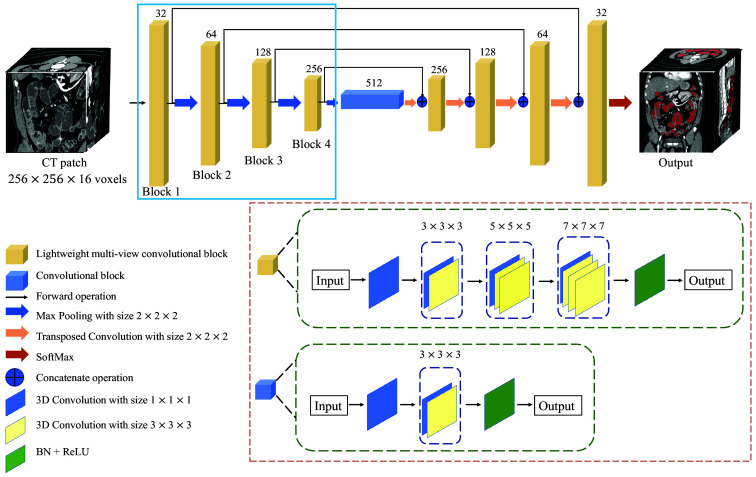
Structure of LMVS-Net. BN denotes batch normalization, and ReLU denotes the ReLU activation function. The numbers above the lightweight multi-view convolutional blocks indicate the number of kernels in each convolutional block. The detailed structure of the lightweight multi-view convolutional block and the convolutional block is shown in the red box. The encoder is shown in the blue box, and we show the internal feature map extracted by Blocks 1–4 in Fig. 4.

**Fig. 4:**
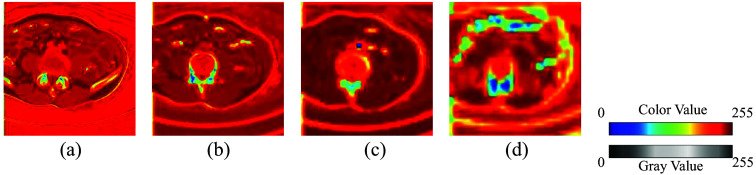
Internal feature map visualization extracted by the encoder of LMVS-Net. Panels (a)–(d) are feature maps extracted by each convolutional block. Regions with large feature values are colored in red, and lower values in yellow or blue. Intestinal regions tend to be colored in yellow or blue. Blocks 1 (a), 2 (b), 3(c), and 4 (d) mean the convolution block in the encoder part.

Szegedy et al.^23^ introduced Inception-v2 using convolutional kernels of different sizes to capture multi-scale features enabling the network to fully utilize features from multi-view, which helps enhance the network’s learning ability. Furthermore, Inception-v2 uses small-size convolutional kernels to reduce the number of parameters, making the network more feasible for training. We integrate concepts from Inception-v2 into the 3D U-Net, leveraging multi-scale features to enhance network performance. Considering that CT volumes are 3D images requiring significant computational resources, we also employ stacked small-size convolutional kernels to achieve the effect of large-size convolutional kernels, which reduce computational costs while preserving a symmetric structure. Specifically, we use two  3×3×3 convolution kernels to realize the convolution effect of one  5×5×5, and three  3×3×3 convolution kernels to realize the convolution effect of one  7×7×7. The  1×1×1 convolution kernel in the multi-view convolutional block is designed to change the number of channels and decrease the parameters of the model. The detailed structure of the LMVS-Net is shown in Fig. 3. First, we get the mean of the feature maps according to the number of channels and output the final feature maps. Second, we normalized the gray of feature maps to (0-255). Finally, we use the color mapping method^24^ in OpenCV to map the feature maps’ gray values to color values from blue to red. In our research, we used blue to red. The visualization in Fig. 4 shows that the intestine regions are more obvious as the deeper of the convolutional block.

### 2.3 Bidirectional Teaching

**Fig. 5:**
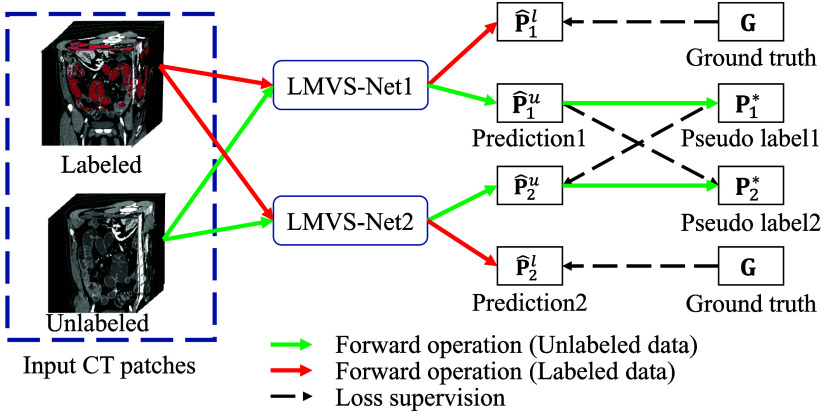
Overview of the bidirectional teaching between two LMVS-Nets. It is worth noting that the input for each iteration is a pair of labeled and unlabeled patches. Pseudo-labels are generated for unlabeled data, enabling the utilization of large-scale unlabeled data during training.

We train two LMVS-Nets having different initial weights using CT patches $\;X^{l}$ with their ground-truth G and unlabeled CT patches $X^{u}$ as input. The overview of bidirectional teaching between two LMVS-Nets is shown in Fig. 5. The input consists of a pair of labeled and unlabeled data, prompting the proposed framework to generate two corresponding predictions, P^il=fi(Xl) and P^iu=fi(Xu) from one LMVS-Net, where i∈{1,2} denotes the i’th LMVS-Nets (LMVS-Net1, LMVS-Net2), as shown in Fig. 5, $\;f_{i}(\cdot )$ represents i’th LMVS-Net. P^il and P^iu denote the predictions from the labeled and the unlabeled CT patches, respectively. We obtain the pseudo-labels by binarizing the predictions using the argmax operation. The LMVS-Net1 generates the pseudo-label for the prediction of unlabeled CT patches from LMVS-Net2, and vice versa

$$P_{i}^{*}=\arg\;\max(P_{i}^{u}),$$ (1)

where $P_{i}^{*}$ represents the pseudo-labels. The two LMVS-Nets supervise each other until training is complete, called bidirectional teaching.

### 2.4 Loss Function with Distance Weight

The overall loss is a compound loss with two parts, a supervised loss, $L_{\text{sup}}$, for the labeled data, and an unsupervised loss, $L_{\mathrm{un}}$, for the unlabeled data. The supervised loss consists of two loss functions

Lsup(P^il,G)=αLdice(P^il,G)+(1−α)Lcldice(P^il,G), (2)

where $L_{\text{D}\text{ice}}$ denotes Dice loss, which is commonly used in medical image segmentation. $L_{\text{cldice}}$ denotes clDice loss,^21^ which uses the intersection between the prediction’s centerline and the ground truth and the intersection of the prediction and the ground truth’s centerline to calculate the loss value. P^il denotes the prediction of labeled CT patches in i’th LVSM-Net (i∈{1,2}), and G denotes the ground truth. α denotes the weight of the loss function, and we experimentally set the value of α=0.5.

In the unsupervised loss function, pseudo-labels are assigned to unlabeled data, and we calculate the loss. However, these pseudo-labels are derived from the model’s predictions and may be unreliable. It is crucial to handle the pseudo-labels carefully to reduce the influence of the unreliable. As we all know, the segmentation in the boundary part is a common challenge in medical image segmentation tasks. In this work, we calculate the mean of the distance maps for each patch and use it as a weight in the loss function. This allows us to assign higher weights to patches that contain fewer boundary regions. Specifically, we generate a distance map by Euclidean distance transformation^25^ to calculate the distance map. Then, we normalize the distance map to [0,1] by the min–max normalization. Finally, we use the mean of the normalized distance map as a weight, called distance weight, and assign the weight to the unsupervised loss. Consequently, the unsupervised loss is defined as follows

Lun(P^iu,Pj*,Dj)=1XYZ∑x=0X−1∑y=0Y−1∑z=0Z−1Ldice(P^iu,Pj*)Dx,y,zj, (3)

where P^iu denotes the prediction of unlabeled CT patches in i’th LMVS-Net, and $P_{j}^{*}$ denotes pseudo-labels from j’th LMVS-Net. Note that i∈{1,2}, j∈{1,2}, and i≠j. $\;D^{j}$ represent the normalized distance map generated from $P_{j}^{*}$, and X, Y, and Z denote the number of voxels in each of the three dimensions of $\;D^{j}$. x∈{0,…,X−1}, y∈{0,…,Y−1}, and z∈{0,…,Z−1} denote the position of each voxel. $\;D_{x,y,z}^{j}$ denotes the value of $\;D^{j}$ at position (x,y,z).

The final loss L is defined as

L=Lsup(P^1l,G)+Lsup(P^2l,G)+γLun(P^1u,P2*,D2)+γLun(P^2u,P1*,D1), (4)

where γ is a weight factor, which is defined by the training iteration, $\gamma (t)=0.1e^{\omega }$,

$\;\omega =-5(1-\frac{t}{T})$ and t=(1,2,…,T) denote the current iteration, and T represents the total iteration number.

## 3 Experiments and Results

### 3.1 Dataset and Evaluation Metrics

In this work, we used a dataset consisting of 171 cases of ileus patients’ CT volumes and introduced detailed information on CT volumes in Table 1. The ground-truth labels were annotated by a technical researcher, two medical students, and a surgeon. The labels annotated by the technical researcher and the medical students were reviewed by two surgeons, including the surgeon working on annotation. Both surgeons’ experience was 8 years.

**Table 1:** Detailed information on CT volumes after interpolation. Information of CT volumes after interpolation. We present the slice size, slice number, pixel spacing, and slice thickness of our intestine dataset.

	Original	Interpolation result
Slice size (pixels)	512 × 512	(281 − 463) × (281 − 463)
Slice number (slices)	198 − 546	396 − 762
Resolution (mm)	(0.549 − 0.904) × (0.549 − 0.904) × (1.000 − 2.000)	1.000 × 1.000 × 1.000

We used 85 CT volumes for training, including 13 densely labeled and 72 unlabeled CT volumes. Then, 27 CT volumes with sparsely labeled were used for validation, 59 CT volumes including 58 with sparsely labeled, and one with densely labeled for testing. Here, the densely labeled CT volume means the clinicians labeled intestines in hundreds of continuous slices but did not label intestine regions in every slice. The sparsely labeled CT volume means the clinicians labeled intestines in some of the discontinuous slices.

Before training, we cropped the 3D CT patches with 256×256×16  voxels to trade off the computational efficiency. To enhance the generalization ability of the segmentation model, we employed data augmentation in our experiments, including flipping and cut-out. We quantitatively evaluated the segmentation results using three accuracy metrics: (1) Dice, (2) recall, (3) precision rates, and (4) one distance-based metric: normalized surface distance^26^ (NSD), also called normalized surface Dice. Almost all of the testing cases were sparsely labeled; for one sparsely labeled CT volume, the percentage of labeled slices in each CT volume ranged from 1.00% to 5.31%, with the number of labeled slices varying between 6 and 29. In the evaluation, we infer CT volumes and get 3D segmentation. However, we extracted the 2D-labeled CT slice to calculate the validation metrics. The total number of 2D CT slices was 1202. Furthermore, we repeated every experiment five times with different random seeds, which can prove that our method had good performance even with different initializations. We calculated the average accuracy metrics of 59 test cases in each experiment and calculated the standard deviation (SD) of the metrics of the five experiments.

### 3.2 Implementation Details

In this work, our proposed method was implemented using PyTorch and executed on an NVIDIA A100 80G GPU. The model was trained using the stochastic gradient descent (SGD) optimizer with a batch size of 8. The initial learning rate was set to 0.01, and the poly learning rate strategy was used to adjust the learning rate. In addition, the number of iterations was set to 30,000, and the model obtained from the final iteration was used for the prediction.

### 3.3 Results

To validate the proposed method’s effectiveness, we conducted a comparison with different methods, 3D U-Net,^26^ CPS,^13^ EM,^18^ and MT.^17^ All these methods use 3D U-Net as the backbone. Table 2 shows the segmentation performances of these methods on the intestine segmentation dataset. It can be seen that the CPS method achieved a Dice score of 76.00%, precision of 84.64%, and recall of 73.97%. The proposed method achieved the best Dice score of 80.45%, precision of 84.12%, and recall of 78.84%. For the distance-based metric, NSD, the proposed method outperforms others when using 13 or six labeled cases. The Dice score measures volumetric overlap, whereas NSD focuses on boundary alignment within a tolerance threshold. In the experiment, we set the tolerance to 3.0 mm. As shown in Table 2, the NSD values for all methods are lower than their corresponding Dice scores. This difference arises because the boundary of the intestines is complex and irregular, making segmentation more challenging in the boundary regions. We calculated the p-value to assess the validity of our method using the Wilcoxon signed-rank test on the Dice scores. The evaluation was conducted when the training dataset contained 13 densely labeled cases and 72 unlabeled cases. In the test, we obtained p-values (0.000, 0.002, 0.000, 0.004) that were all <0.05 between the Dice scores of the proposed method and four existing methods (3D U-Net, CPS, EM, and MT) based on the Wilcoxon signed-rank test. The result indicated the validity of our method. In addition, the proposed method uses stacks of small-sized convolutional kernels instead of large convolutional kernels to reduce the model’s parameter. The proposed model, using stacks of small-sized convolutional kernels instead of large convolutional kernels, has 14M parameters (Dice score 80.45%). In comparison, the model^19^ employing only large convolutional kernels has 17M parameters (Dice score 78.86%). 3D U-Net^26^ has 19M parameters. Compared with the 3D U-Net, the proposed method also has fewer parameters.

**Table 2:** We compared the quantitative results of the proposed method with four previous methods, including the classical fully supervised method (3D U-Net) and three semi-supervision methods (CPS, EM, and MT) when the model was trained using 6 cases and 13 cases as densely labeled samples.

Labeled	Methods	Dice (%)	Precision (%)	Recall (%)	NSD (%)
6 Cases	3D U-Net^26^	46.20±7.10	59.28±5.47	44.57±10.77	16.50
	CPS^13^	72.99±1.00	84.26±1.22	67.63±2.13	29.07
	EM^18^	69.45±2.05	84.39±1.06	62.50±3.38	28.54
	MT^17^	69.73±6.26	82.48±1.21	63.78±8.46	28.09
	LMVS-Net (Proposed)	77.18±1.87	84.42±1.33	73.19±3.39	**31.66**
13 Cases	3D U-Net	$46.80^{⋆}\pm 13.96$	80.75±3.77	37.80±17.88	20.62
	CPS	$76.00^{⋆}\pm 3.84$	84.64±0.69	71.79±0.516	31.97
	EM	$76.10^{⋆}\pm 2.84$	85.40±1.33	71.19±4.96	31.11
	MT	$74.05^{⋆}\pm 6.82$	86.17±1.03	68.20±9.63	31.44
	LMVS-Net (Proposed)	80.45±0.71	84.12±0.98	78.84±1.76	**33.50**

Given an evaluation metric, the null hypothesis of our test is that there is no significant difference in our method outperforming other methods in terms of the metric. We set the significance level at 0.05. We conducted the Wilcoxon signed-rank test on the Dice scores when the model was trained using 13 cases as densely labeled data and 72 cases as unlabeled data. In the table, ⋆ denotes p-value <0.05, indicating that our method outperformed the other methods.

The p-values were calculated between the proposed method and four previous methods (3D U-Net, CPS, EM, and MT) on the Dice score. We present the SD and highlight the best performance of each evaluation term with a bold font.

**Fig. 6:**
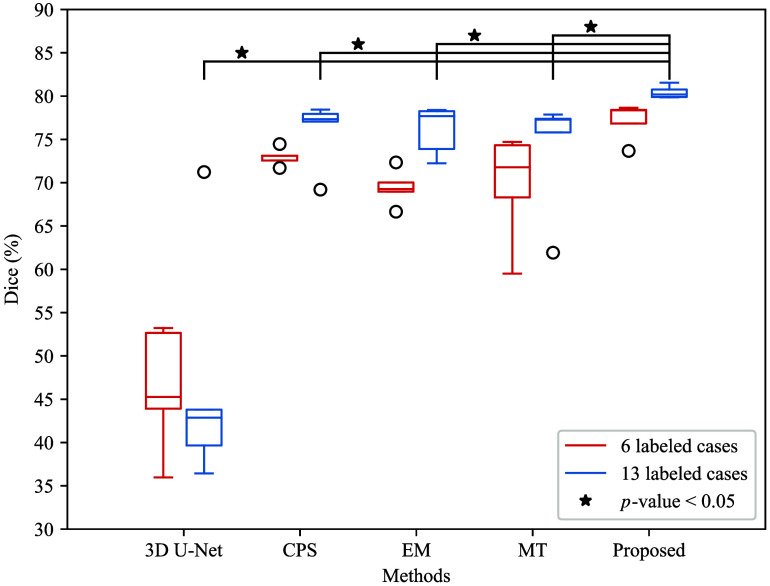
Boxplot of Dice scores of different methods that were trained on datasets containing 6 and 13 densely labeled cases and 72 unlabeled cases. We calculated the p-value using the Wilcoxon signed-rank test on Dice scores when the model was trained on datasets containing 13 densely labeled cases and 72 unlabeled cases, and ⋆ denotes p-value <0.05. The red colors indicate the results when we trained the model using 6 labeled cases and 72 unlabeled cases. The blue colors indicate the results when we trained the model using 13 labeled cases and 72 unlabeled cases. The circle indicates outliers.

**Fig. 7:**
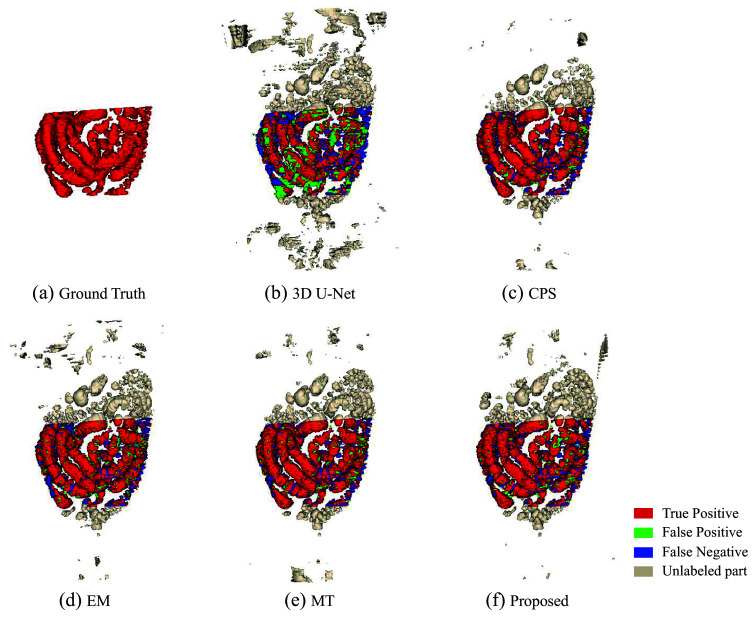
3D intestine segmentation results from different methods. (a) Ground truth; (b)–(e) results of four previous methods; (f) result of the proposed method.

**Fig. 8:**
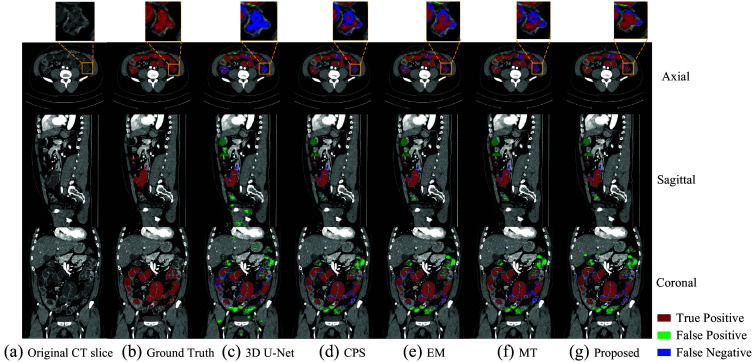
Intestine segmentation results from different methods on axial, sagittal, and coronal three 2D planes. Panel (a) is the original CT slice; panel (b) is the ground truth; panel (c) is the result of 3D U-Net; panel (d) is the result of CPS; panel (e) is the result of EM; panel (f) is the result of MT; and panel (g) is the result of the proposed method.

**Fig. 9:**
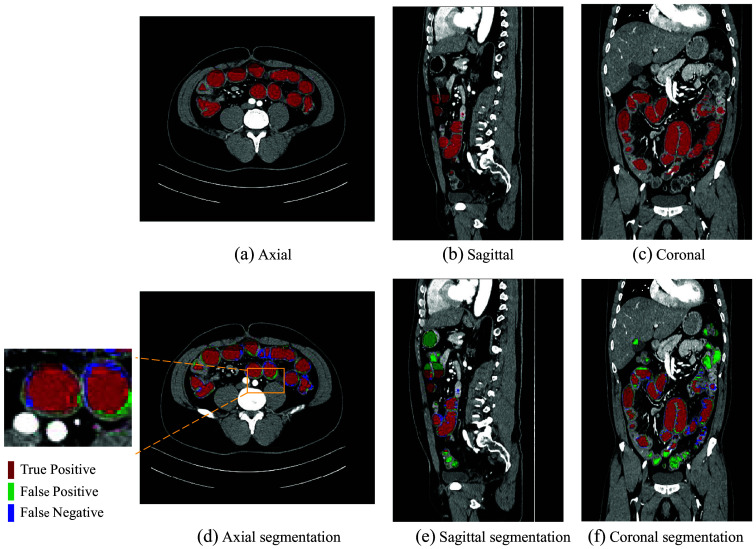
Proposed method’s intestine segmentation results are displayed on three planes: (a), (d) axial; (b), (e) sagittal; (c), (f) coronal. The first row (a)–(c) represents the ground truth, and the second row (d)–(f) displays the corresponding segmentation results.

Figure 6 shows a boxplot according to the Dice scores of different methods trained with 6 and 13 densely labeled cases. The 3D segmentation results of these methods are illustrated in Fig. 7. In Fig. 7, the regions with red color indicate true positives, the regions with green color indicate false positives, and the regions with blue color indicate false negatives. As we use a densely labeled case to present the 3D visualization result, intestine regions lack labels in certain slices. However, these methods can effectively segment unlabeled intestine regions, depicted in gray. We use gray to indicate these unlabeled intestine regions. Figure 8 shows the 2D intestine segmentation results in axial, sagittal, and coronal three planes by different methods. The segmentation results of the proposed method from three different planes are shown in Fig. 9.

We also compared the proposed with two previous methods for intestine segmentation (M U-Net, MVS-Net) and show the result in Table 3. M U-Net^27^ is an improved 3D U-Net segmentation model based on full-supervision learning, which can utilize the multi-dimensional features from CT volumes. MVS-Net^19^ is a semi-supervised method similar to the proposed method. We can see from the result that our method achieves the highest Dice and the NSD values, and M U-Net, an improved 3D U-Net segmentation model based on fully supervised learning, achieves the lowest Dice and NSD.

**Fig. 10:**
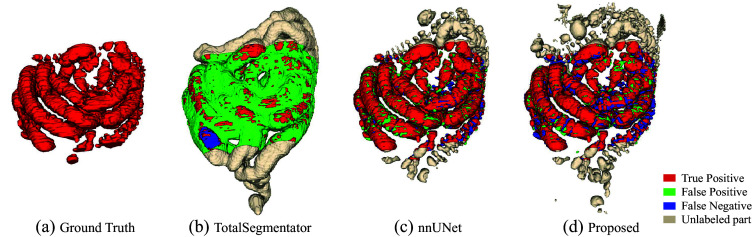
Results of TotalSegmentator, nnUNet, and our method. (a) Ground truth. (b) TotalSegmentator. (c) nnUNet. (d) Proposed.

**Fig. 11:**
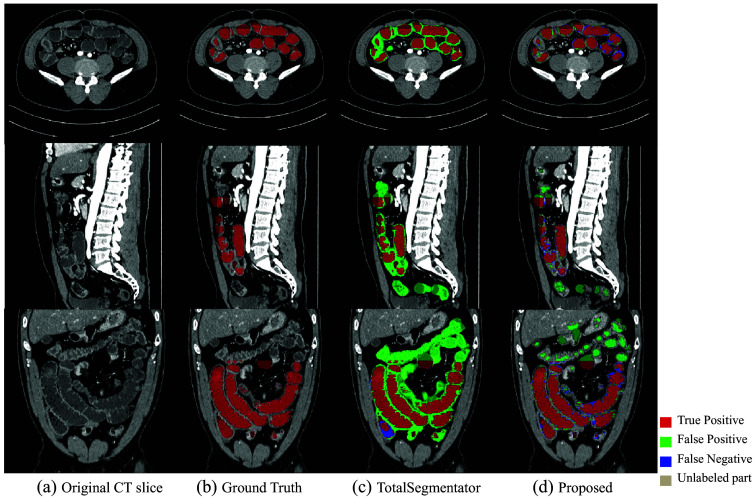
2D visualization segmentation result of TotalSegmentator and the proposed method. (a) Original CT slice. (b) Ground truth. (c) TotalSegmentator. (d) Proposed.

In addition, we compare our method with the TotalSegmentator,^28^ the quantitative results shown in Table 4, and the qualitative results shown in Fig. 10 (3D visualization) and Fig. 11 (2D visualization).

**Table 3:** Results of three intestine segmentation methods. We highlight the best performance of each evaluation term with a bold font.

Methods	Dice (%)	Precision (%)	Recall (%)	NSD (%)
M U-Net^27^	73.22±4.94	79.89±6.79	70.61±6.65	27.62
MVS-Net^19^	78.50±8.06	85.88±8.34	75.06±8.46	28.30
LMVS-Net (Proposed)	80.45±0.71	84.12±0.98	78.84±1.76	**31.66**

**Table 4:** Results of TotalSegmentator, nnUNet, and the proposed method. We highlight the best performance of each evaluation term with a bold font.

Methods	Dice (%)	Precision (%)	Recall (%)	NSD (%)
TotalSegmentator^28^	56.56	50.03	78.55	15.50
nnUNet^29^	**81.32**	**84.51**	**79.82**	**36.27**
LMVS-Net (Proposed)	80.45	84.12	78.84	31.66

### 3.4 Ablation Study

**Fig. 12:**
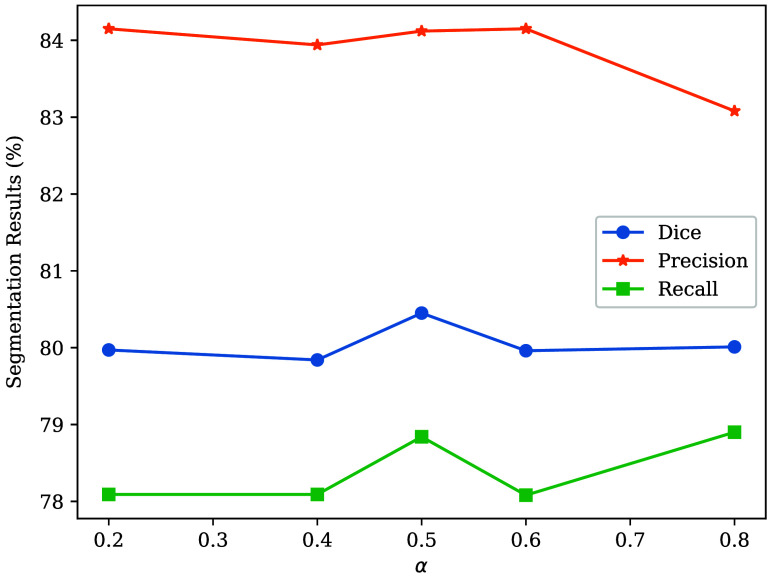
Line chart of different weights (α) in the supervision loss functions, Eq. (2). We can see that the best Dice score is achieved when α=0.5.

Table 5 presents the ablation study result of LMVS-Net. We combine different architectures with bidirectional teaching. The results showed that compared with the 3D U-Net and attention U-Net, the LMVS-Net achieved the best performance when combined with bidirectional teaching. The result of the ablation study of the loss function is shown in Table 6. We used the LMVS-Net with different loss functions to train the segmentation model. It can be found that compared with only using Dice loss, clDice loss performs not very well. However, combining the two loss functions performed well in the intestine segmentation task. As the Dice loss focuses on overall segmentation accuracy, the clDice loss can use critical centerline information to improve performance. We evaluated the influence of weight α in the supervision loss function 2, and the result is shown in Fig. 12. We can find that the best segmentation accuracy is achieved when α=0.5. We evaluate the effectiveness of the distance weight map and show the result in Table 7. We conducted the experiments with and without distance weight and showed the quantitative results. It can be found that the proposed distance weight achieved higher segmentation accuracy, improved 1.50% Dice score, and 3.70% precision.

**Table 5:** To validate the effectiveness of the LVMSNet, we use different models with bidirectional teaching (BT). We highlight the best performance of each evaluation term with a bold font.

Method	Dice (%)	Precision (%)	Recall (%)
D U-Net with BT	76.00±3.84	84.64±0.69	71.79±5.16
3D U-Net and ATT U-Net with BT	75.80±2.67	85.25±1.48	71.02±4.73
ATT U-Net with BT	71.55±1.87	85.80±0.56	64.62±2.57
LMVS-Net with BT	78.11±1.30	85.63±1.11	73.66±2.59

**Table 6:** Results of the ablation studies for the loss functions. We use different loss functions in the proposed method. We highlight the best performance of each evaluation term with a bold font.

Method	Dice (%)	Precision (%)	Recall (%)
LMVS-Net with Dice	79.14±1.79	84.52±0.88	76.25±3.09
LMVS-Net with clDice	78.33±1.99	85.05±1.01	74.56±2.59
LMVS-Net with CE and Dice	78.11±1.30	85.63±1.11	73.66±2.59
LMVS-Net with clDice and Dice	79.42±1.10	84.78±1.43	76.58±2.68

**Table 7:** Ablation study of the distance weight (DW). We highlight the best performance of each evaluation term with a bold font.

Method	Dice (%)	Precision (%)	Recall (%)
LMVS-Net without DW	79.66±2.11	85.17±1.24	76.78±4.58
LMVS-Net with DW	80.45±0.71	84.12±0.98	78.84±1.76

## 4 Discussion

Our proposed method segmented most of the intestine regions when we trained it with labeled and unlabeled data. Table 2 shows the results of four previous methods and the proposed method on our dataset. Compared with the full-supervision method 3D U-Net, the proposed method and other semi-supervision methods can use the information from unlabeled data and achieve better segmentation results, which denoted that the use of unlabeled data helps reduce the influence of limited labeled data in the intestine segmentation task. Compared with EM and MT the two semi-supervision methods, CPS and the proposed method achieved better performances. EM focuses on making the model more certain about its predictions of unlabeled data and MT focuses on enhancing the model’s consistency and reducing uncertainty in its predictions of unlabeled data. However, the CPS and the proposed method use the bidirectional teaching strategy and assign pseudo-labels to the unlabeled data to improve model performance. We also investigated the performance between CPS and the proposed method. The CPS method first proposes bidirectional teaching between two 3D U-Nets. The proposed method optimizes the original CPS’s backbone, replacing the 3D U-Net with LMVS-Net, and uses bidirectional teaching between two LMVS-Nets. The LMVS-Net achieves multi-scale semantic information from various perceptual fields, which effectively improves the model’s segmentation performance.

Furthermore, we explore the model’s performance when the model was trained using six cases as labeled data and 72 unlabeled cases as unlabeled data, as well as 13 cases as labeled and the same 72 unlabeled cases as unlabeled data. It can be found that the Dice score decreases when we reduce the number of labeled data in the training dataset. We repeated the experiment five times for each method. Figure 6 shows the distribution of five repeated experiments’ Dice scores for each method. In the figure, we can find that the proposed method achieves the best Dice score.

Figure 7 shows the qualitative results between various methods when the training dataset contains 13 labeled CT volumes and 72 unlabeled CT volumes. The proposed method can segment more intestine regions with fewer mis-segmentation regions than other methods, especially for 3D U-Net. We can see more detailed results in Fig. 8. The figure shows the intestine segmentation results of different methods in axial, sagittal, and coronal planes, respectively. It can be found that the proposed method can segment more intestine regions with fewer false positives such as the regions in yellow boxes. Figure 9 shows the visualization results of the proposed method in three planes. The figure shows that there is still some mis-segmentation in the boundary regions. The reason may be that the intestine exhibits significant shape variations and low contrast compared with surrounding tissues, which presents more challenges.

Compared with TotalSegmentator, our method achieves higher accuracy in intestine segmentation. TotalSegmentator is a pre-trained model designed to segment various organs, including the entire intestinal region, not just the intestinal lumen. As a result, it exhibited lower precision when applied to our testing dataset. The experimental results demonstrate that when the method trained based on a large dataset is transferred to our task, the results are not very good due to the domain gap problem. Our method is more competitive for the intestine segmentation task. nnUNet employs various performance optimization strategies, including hyperparameter tuning and data augmentation, which automatically adjust numerous parameters to select the optimal settings for segmentation tasks. By contrast, our method uses fixed settings. In terms of model size, nnUNet (88M parameters) is significantly larger than our proposed method (14M parameters). Regarding accuracy, we hypothesized that there would be no significant difference between nnUNet and our method and set the significance level at 0.05. Using the Wilcoxon signed-rank test, we calculated a p-value >0.05, indicating no significant difference in performance between the two methods. Although our method is less accurate compared with nnUNet, it uses less than a quarter of the parameters. Thus, we can conclude that achieving over 80% Dice score with fewer parameters is a noteworthy success for our approach.

## 5 Conclusion

This paper proposed an intestine segmentation method, which is based on the LMVS-Net incorporated with bidirectional teaching for intestine segmentation from CT volumes. The proposed method aims to effectively utilize large-scale unlabeled data and important centerline information of tubular organs. Furthermore, the method decreases the influence of pseudo-labels’ unreliability by generating weights based on their distance maps. The proposed method demonstrates good segmentation performance and offers competitive results compared with the baseline and previous semi-supervision methods. We compared the segmentation results of several previous methods, including 3D U-Net, EM, and MT. In these methods, the 3D U-Net is a full-supervision method that relies on a substantial amount of labeled data, making it not very well compared with the semi-supervision methods for intestine segmentation tasks.

Although significant progress has been made in intestinal segmentation for the proposed method, there is still some space to improve the segmentation accuracy in the boundary part. In future research, we aim to utilize advanced clinical experiments as prior knowledge to reduce annotation costs, particularly for intestine segmentation. The task often involves complicated spatial structures and belongs to the dense-label-based tasks. By effectively utilizing such prior knowledge, we can enhance the accuracy and efficiency of the segmentation. Furthermore, solving the domain gap problem to transfer the model trained by a large dataset to the intestine segmentation tasks and integrating optimization techniques similar to nnUNet in our method to produce higher performance with semi-supervision also deserve investigation.

## Data Availability

The code can be found at: https://github.com/MoriLabNU/semi-pseudo-labels. The dataset mentioned in the paper is private and has been ethically reviewed. The IRB Approval Number of the dataset is Nagoya University School of Medicine IRB Approval Number 2017-0103.
